# Symmetry-Based Representations for Artificial and Biological General Intelligence

**DOI:** 10.3389/fncom.2022.836498

**Published:** 2022-04-14

**Authors:** Irina Higgins, Sébastien Racanière, Danilo Rezende

**Affiliations:** DeepMind, London, United Kingdom

**Keywords:** machine learning, representation learning, symmetries, physics, neuroscience, vision

## Abstract

Biological intelligence is remarkable in its ability to produce complex behavior in many diverse situations through data efficient, generalizable, and transferable skill acquisition. It is believed that learning “good” sensory representations is important for enabling this, however there is little agreement as to what a good representation should look like. In this review article we are going to argue that symmetry transformations are a fundamental principle that can guide our search for what makes a good representation. The idea that there exist transformations (symmetries) that affect some aspects of the system but not others, and their relationship to conserved quantities has become central in modern physics, resulting in a more unified theoretical framework and even ability to predict the existence of new particles. Recently, symmetries have started to gain prominence in machine learning too, resulting in more data efficient and generalizable algorithms that can mimic some of the complex behaviors produced by biological intelligence. Finally, first demonstrations of the importance of symmetry transformations for representation learning in the brain are starting to arise in neuroscience. Taken together, the overwhelming positive effect that symmetries bring to these disciplines suggest that they may be an important general framework that determines the structure of the universe, constrains the nature of natural tasks and consequently shapes both biological and artificial intelligence.

## 1. Introduction

Neuroscience and machine learning (ML) have a long history of mutually beneficial interactions (Hassabis et al., 2017), with neuroscience inspiring algorithmic and architectural improvements in ML (Rosenblatt, 1958; LeCun et al., 1989), and new ML approaches serving as computational models of the brain (Yamins et al., 2014; Yamins and DiCarlo, 2016; Wang et al., 2018; Dabney et al., 2020). The two disciplines are also interested in answering the same fundamental question: what makes a “good” representation of the often high-dimensional, non-linear, and multiplexed sensory signals to support general intelligence (Bengio et al., 2013; Niv, 2019). In the same way as the adoption of the decimal system for representing numbers has produced an explosion in the quantity of numerical tasks that humans could solve efficiently (note that the information content remained unaffected by this change in the representational form), finding a “good” representation of the sensory inputs is likely to be a fundamental computational step for enabling data efficient, generalizable, and transferrable skill acquisition. While neuroscientists go about trying to answer this question by studying the only working instantiation of general intelligence—the brain, ML scientists approach the same problem from the engineering perspective, by testing different representational forms in the context of task learning through supervised or reinforcement learning (RL), which allows faster iteration. In this review we will discuss how bringing the idea of symmetry transformations from physics into neural architecture design has enabled more data efficient and generalizable task learning, and how this may be of value to neuroscience.

The reason why it makes sense to turn to physics when it comes to understanding the goal of perception in artificial or biological intelligence, is because intelligence evolved within the constraints of our physical world, and likewise, the tasks that we find interesting or useful to solve are similarly constrained by physics. For example, it is useful to know how to manipulate physical objects, like rocks, water or electricity, but it is less useful to know how to manipulate arbitrary regions of space (which also do not have a word to describe them, further highlighting their lack of relevance). Hence, a representation that reflects the fundamental physical properties of the world is likely to be useful for solving natural tasks expressed in terms of the same physical objects and properties. *Symmetry transformations* are a simple but fundamental idea that allows physicists to discover and categorize physical objects—the “stubborn cores that remain unaltered even under transformations that could change them” (Livio, 2012), and hence symmetries are a good candidate for being the target of representation learning.

The study of symmetries in physics (that is, the transformations that leave the physical “action” invariant) in its modern form originates with Noether's Theorem (Noether, 1915), which proved that every conservation law is grounded in a corresponding continuous symmetry transformation. For example, the conservation of energy arises from the time translation symmetry, the conservation of momentum arises from the space translation symmetry, and the conservation of angular momentum arises due to the rotational symmetry. This insight, that transformations (the joints of the world) and conserved properties (the invariant cores of the world that words often tend to refer to Tegmark, 2008) are tightly related, has led to a paradigm shift in the field, as the emphasis in theoretical physics changed from studying objects directly to studying *transformations* in order to discover and understand objects. Since the introduction of Noether's theorem, symmetry transformations have permeated the field at every level of abstraction, from microscopic quantum models to macroscopic astrophysics models.

In this paper we are going to argue that, similarly to physics, a change in emphasis in neuroscience from studying representations in terms of static objects to studying representations in terms of what natural symmetry transformations they reflect can be impactful, and we will use the recent advances in ML brought about by the introduction of symmetries to neural networks to support our argument. By introducing the mathematical language of group theory used to describe symmetries, we hope to provide the tools to the neuroscience community to help in the search for symmetries in the brain. While ML research has demonstrated the importance of symmetries in the context of different data domains, here we will mainly concentrate on vision, since it is one of the most prominent and most studied sensory systems in both ML and neuroscience. For this reason, topics like the importance of symmetries in RL will be largely left out (although see Agostini and Celaya, 2009; Anand et al., 2016; Madan et al., 2018; van der Pol et al., 2020; Kirsch et al., 2021). We will finish the review by describing some of the existing evidence from the neuroscience community that hints at symmetry-based representations in the ventral visual stream.

## 2. What Are Symmetries?

### 2.1. Invariant and Equivariant Representations

Given a task, there often exist transformations of the inputs that should not affect it. For example, if one wants to count the number of objects on a table, the outcome should not depend on the colors of those objects, their location or the illumination of the scene. In that case, we say the output produced by an intelligent system when solving the task is *invariant* with respect to those transformations. Since the sensory input changes with transformations, while the output is invariant, we need to decide what should happen to the intermediate representations. Should they be invariant like the output or should they somehow transform similarly to the input?

**Figure 1:**
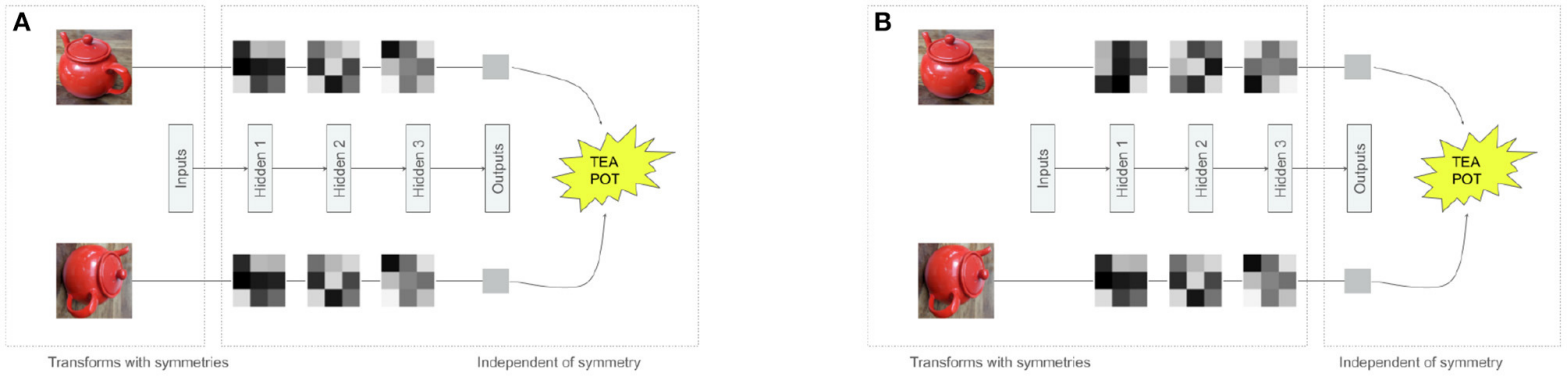
Different approaches to dealing with symmetries in neural networks. Both figures represent a neural network transforming an image, and a rotated image. The gray 3 × 3 squares are activations of the neural networks. **(A)** Inputs transform with symmetries, but hidden features and outputs are invariant. **(B)** Inputs and hidden features transform with symmetries, only outputs are invariant.

**Figure 2:**
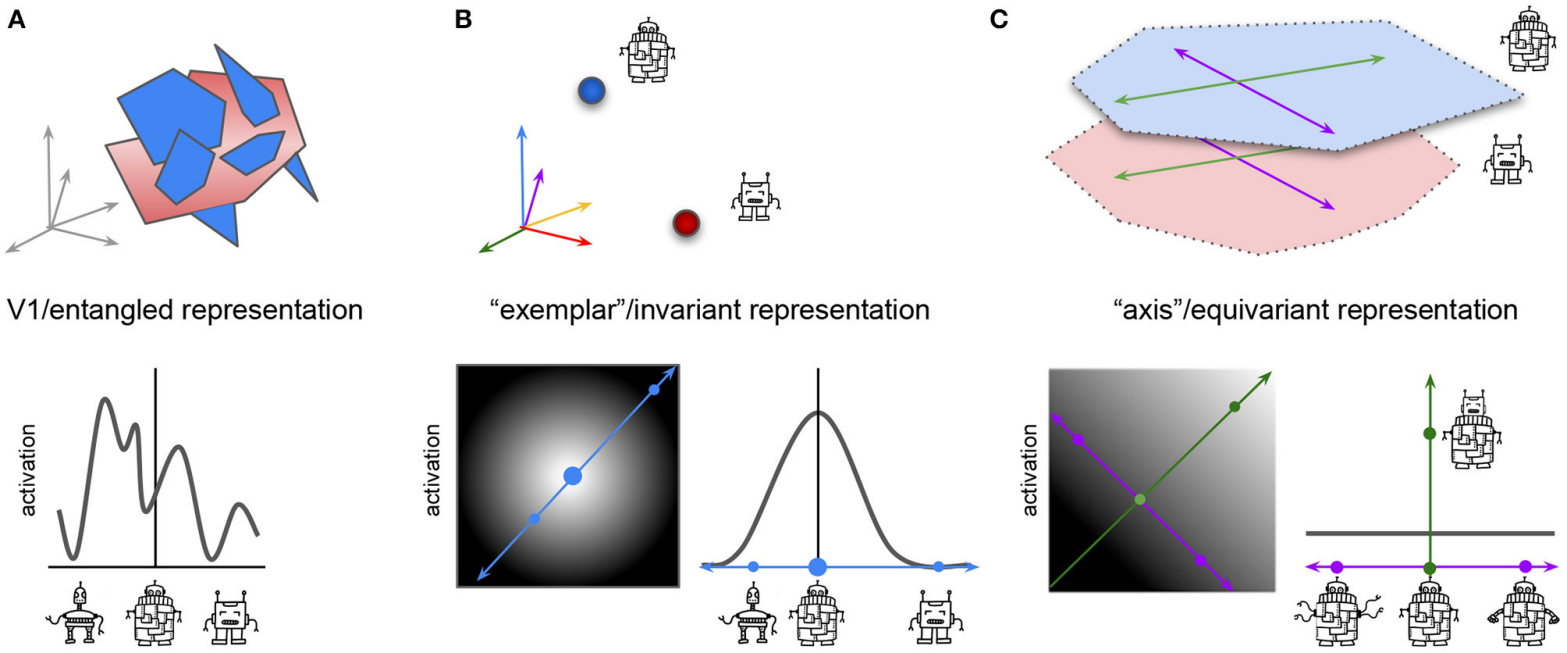
Different hypothesized coding properties of neurons at the start **(A)** and end **(B,C)** of visual processing in neural networks and the brain. Top row, schematic representation of manifolds representing two classes of robots: blue manifold contains robots that vary in the shape of their head, arms, legs, and body; red manifold contains robots that have no body and vary in the shape of their head, arms and legs only. Bottom row, schematic representation of the activations of a single idealized real or artificial neuron in response to variations in the visual stimulus. **(A)** (Top) Early processing stages have entangled high-dimensional manifolds. All information about the two robot categories, and their identity preserving transformations is present, but is hard to read out from the neural activations. Arrows represent the high-dimensional space spanned by all V1 neurons. (Bottom) Line plot shows the activation of a single idealized neuron in response to different robot variations. Neuron responds to robots from both classes. **(B)**: “Exemplar” or invariant representation at the end of visual processing. Top: Single neurons have maximal firing for the prototype example of their preferred robot class (blue—with, red—without body). All information about the identity preserving transformations has been collapsed (illustrated by red or blue points), which makes object classification easy, but any other task (like clustering robots based on their arm variation) impossible. Arrows represent the high-dimensional space spanned by the higher-order neurons, arrow color represents preferred stimulus of the neuron. Bottom left: activation of a single idealized neuron to all robot variations from both classes. Lighter, higher activation. Big blue circle indicates preferred stimulus for neuron, resulting in highest activation, smaller blue circles indicate other robots resulting in lower to no activation. Blue arrow, cross section of robot variations shown in the line plot. Bottom right: line plot shows activation of the same idealized neuron as on the left but in the cross section of robot variations spanned by the blue arrow. Response declines proportionally to the distance from the preferred stimulus (big blue circle). **(C)** “Axis” or equivariant representation at the end of visual processing. Top: two robot classes have been separated into different representational manifolds, which are also aligned in terms of the shared transformations (e.g., both robot classes have similar identity preserving transformations in head shape, spanned by green axis; and arm shape, spanned by purple axis). This makes it easy to classify the robots, and solve other tasks, like clustering robots based on their arm variations. Bottom left: activation of a single idealized neuron to robot variations along the head shape change axis (green) and arm shape change axis (purple). Lighter, higher activation. Neuron has a ramped response proportional to changes in its preferred transformation (changes in head shape, green), and no change in firing to other transformations (e.g., changes in arm shape, blue). Bottom right: as in **(B)**, but the cross section is spanned by the purple axis. Green dot indicates higher neural activation in response to a change in the robot head shape.

Much of the research on perception and representation learning, both in ML and neuroscience, has focused on object recognition. In ML, this line of research has historically emphasized the importance of learning representations that are *invariant* to transformations like pose or illumination (Lowe, 1999; Dalal and Triggs, 2005; Sundaramoorthi et al., 2009; Soatto, 2010; Krizhevsky et al., 2012). In this framework, transformations are considered nuisance variables to be thrown away (Figures 1A, 2B). Some of the most successful deep learning methods (Krizhevsky et al., 2012; Mnih et al., 2015; Silver et al., 2016; Espeholt et al., 2018; Hu et al., 2018; Dai et al., 2021) end up learning such invariant representations (see Tishby et al., 1999; Tishby and Zaslavsky, 2015 for a potential explanation of why this happens in the context of supervised learning). This is not a problem for narrow intelligence, which only needs to be good at solving the few tasks it is explicitly trained for, however, discarding “nuisance” information can be problematic for general intelligence which needs to reuse its representations to solve many different tasks, and it is not known ahead of time which transformations may be safe to discard. It is not surprising then that despite the enormous success of the recent deep learning methods trained on single tasks, they still struggle with data efficiency, transfer, and generalization when exposed to new learning problems (Garnelo et al., 2016; Lake et al., 2016; Higgins et al., 2017b; Kansky et al., 2017; Marcus, 2018; Cobbe et al., 2019).

Similarly to ML, in neuroscience ventral visual stream is traditionally seen to be progressively discarding information about the identity preserving transformations of objects (Fukushima, 1980; Tanaka, 1996; Poggio and Bizzi, 2004; Yamins et al., 2014). While neurons in the early processing stages, like V1, are meant to represent all information about the input stimuli and their transformations in high-dimensional “entangled” manifolds, where the identities of the different objects are hard to separate (Figure 2A), later in the hierarchy such manifolds are meant to collapse into easily separable points corresponding to individual recognizable objects, where all the information about the identity preserving transformations is lost, resulting in the so called “exemplar” neurons^1^ following the naming convention of Chang and Tsao (2017). In this view, every neuron has a preferred stimulus identity in response to which the neuron fires maximally, while its response to other stimuli decreases proportionally to their distance from the preferred stimulus (Figure 2B).

An alternative point of view in both disciplines has advocated that instead of discarding information about the identity preserving transformations, information about these factors should be preserved but reformatted in such a way that aligns transformations within the representations with the transformations observed in the physical world (Figures 1B, 2C), resulting in the so called *equivariant* representations (DiCarlo and Cox, 2007; Hinton et al., 2012; Bengio et al., 2013). In the equivariant approach to perception, certain subsets of features may be invariant to specific transformations, but the overall representation is still likely to preserve more overall information than an invariant representation, making them more conducive of diverse task learning (Figure 1B). For example, some hidden units may be invariant to changes in the object color, but will preserve information about object position, while other hidden units may have an opposite pattern of responses, which means that information about both transformations will be preserved across the whole hidden layer, while each individual subspace in the hidden representation will be invariant to all but one transformation. Researchers in both neuroscience and ML communities have independently hypothesized that equivariant representations are likely to be important to support general intelligence, using the terms “untangling” (DiCarlo and Cox, 2007; DiCarlo et al., 2012) and “disentangling” (Bengio, 2009, 2012; Bengio et al., 2013), respectively. We are next going to introduce the mathematical language for describing symmetry transformations and use it to discuss how adding neural network modules that are equivariant to such symmetry transformations can improve data efficiency, generalization, and transfer performance in ML models.

### 2.2. Defining Symmetries and Actions

**Figure 3:**
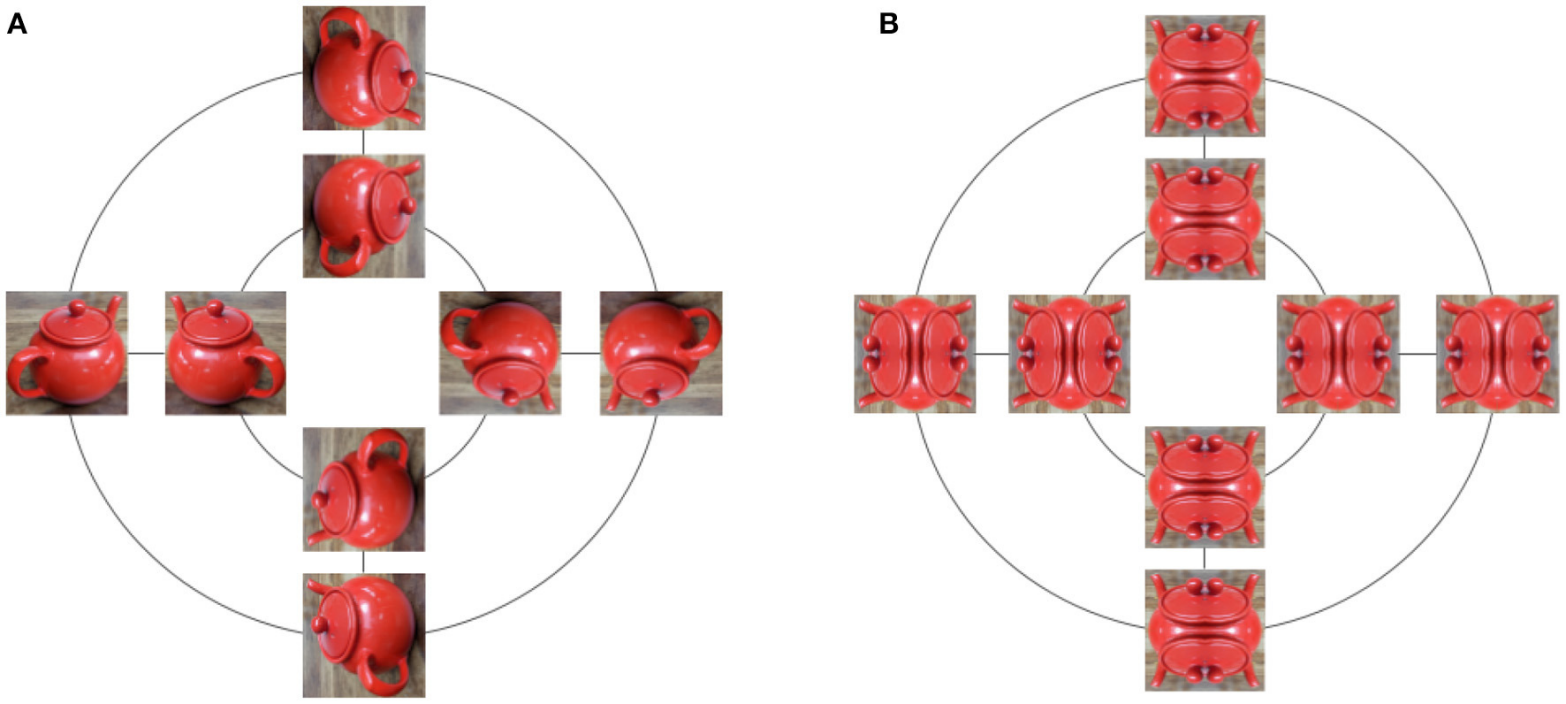
All 8 transformations of an image under the dihedral group *D*_4_. Rotations by 90° are applied along the inner and outer circles. Reflections are applied along straight lines. **(A)** While the image is transformed, some properties, such as the teapot identity or color, are invariant with respect to the applied transformations. **(B)** Symmetric images are left invariant by some elements of *D*_4_, and modified by others.

Symmetries are sets of *transformations* of objects, and the same abstract set of symmetries can transform different objects. For example, consider the set of rotations by multiple of 90° and reflections along both horizontal and vertical axis, known as the dihedral group *D*_4_ (Dummit and Foote, 1991). By rotating images, symmetries from *D*_4_ can be applied to images of cats or tea pots, either 32 × 32 or 1, 024 × 1, 024, color or black and white. In mathematics, the concept of symmetries, that is transformations that are invertible and can be composed, is abstracted into the concept of *groups*. For example, *D*_4_ is a group with eight elements (Figure 3).

More formally, a group *G* is defined as a set with a binary operation (also called composition or multiplication)


(1) $$\begin{array}{l} \;G\times G\to \;G\\ \left(g_{1},g_{2}\right)\mapsto g_{1}\cdot g_{2}, \end{array}$$

such that

1. the operation is associative: (*g*_1_ · *g*_2_) · *g*_3_ = *g*_1_ · (*g*_2_ · *g*_3_);
2. there exists an identity element *e* ∈ *G* such that *e* · *g* = *g* · *e* = *g*, ∀*g* ∈ *G*;
3. all elements are invertible: for any *g* ∈ *G*, there exists *g*^−1^ ∈ *G* such that *g* · *g*^−1^ = *g*^−1^ · *g* = *e*.

Note how we defined a group as a set of symmetries, without explicitly saying what these are symmetries of. That's because the concept of group in mathematics seeks to study properties of symmetries that are independent of the objects being transformed. In practice though, we will of course want to apply symmetries to objects. This is formally defined as an *action*.^2^ For example, the group *D*_4_ can act on both 32 × 32 gray-scale images, that is ℝ^32×32^, and on 1, 024 × 1, 024 color images, that is ℝ^1, 024×1, 024×3^.

More formally, given a group *G* and a set *X*, an action^3^ of *G* on *X* is a map


(2) $$\begin{array}{l} G\times X\to \;X\\ \left(g,x\right)\mapsto g\cdot x, \end{array}$$

such that

1. the multiplication of the group and the action are compatible: *g*_1_ · (*g*_2_ · *x*) = (*g*_1_ · *g*_2_) · *x*;
2. the identity of the group leaves elements of *X* invariant: *e* · *x* = *x*.

Note how we overloaded the symbol · to define both a multiplication in the group, and an action on a set. This makes sense because multiplication of the group defines an action of that group on itself. The identity *e* leaves all elements in *X* invariant *e* · *x* = *x*, but for a given *x*, there can exist *g* ≠ *e* such that *g* · *x* = *x*, for example in Figure 3B.

Two elements of a group are said to *commute* if the order in which we multiply them does not matter. Formally, we say that *g*_1_, *g*_2_ ∈ *G* commute if *g*_1_ · *g*_2_ = *g*_2_ · *g*_1_. If all the elements in the groups commute with each other, the group itself is called commutative.^4^ Even if a group is not commutative, it might still be a product of two subgroups that commute with each other. For example, assume you have three cubes of different sizes and colors, and three pyramids of different sizes and colors. If these objects are put on three different tables, each with a cube and a pyramid, we can move the cubes around while leaving the pyramids where they are, or we can move the pyramids and leave the cubes untouched. The action of re-ordering the cubes is an action of the group of permutations over three elements $S_{3}$. Here we are making that group act on our arrangement of cubes and pyramids, by leaving the pyramids invariant. The action of re-ordering the pyramids is also an action of $S_{3}$. So, overall, we have an action of $S_{3}\times S_{3}$. The group as a whole is not commutative, since each of the $S_{3}$ is not, but it does not matter if we reorder the pyramids first, or the cubes first. Formally, this means that as a set *G* = *G*_1_ × *G*_2_, where *G*_1_ and *G*_2_ are themselves groups, and all elements of *G*_1_ commute with all elements of *G*_2_. This last commutation requirement is important. Indeed, consider once again the case of *D*_4_. Let *F* be the subgroup made of the identity and the reflection along the vertical axis. And let *R* be the group made of rotations by 0, 90, 180, and 270°. Any element of *D*_4_ can be written in a unique way as *f* · *r* for (*f, r*) ∈ *F* × *R*, but since *f* · *r* ≠ *r* · *f*, it is not true that *D*_4_ is equal to *F* × *R* as a group.

We just mentioned the idea that some properties are preserved by symmetries. Indeed, while a group action defines how elements of a set are transformed, it is often useful to also consider what is being preserved under the action. For example, consider a Rubik's cube. Algorithms on how to solve a Rubik's cube use steps described by simple transformations such as “rotate left face clockwise” or “rotate front face anti-clockwise.” The set of all transformations built by such simple rotations of faces forms a group, and that group acts on the Rubik's cube by modifying the colors on faces. But what is being preserved here? The answer is the structure of the cube. Indeed, after any of these transformations, we still have a cube with faces, each made of 9 squares arranged in a regular 3 × 3 grid. In the case of our dihedral group *D*_4_ in Figure 3, colors but also relative distances are being preserved: two pixels in the original image will move to a new location in a rotated image, but their distance from each other is unchanged, thus preserving the object identity.

We are now ready to define the concepts of *invariant* and *equivariant* maps—the building blocks for obtaining the invariant and equivariant representations we introduced earlier. Lets start with invariance. Formally, if a group *G* acts on a space *X*, and if *F* : *X* → *Y* is a map between sets *X* and *Y*, then *F* is invariant if *F*(*g* · *x*) = *F*(*x*), ∀(*g, x*) ∈ *G* × *X*. In words, this means that applying *F* to a point or to a transformed point will give the same result. For example, in Figure 3, the map that recognizes a tea pot in the input picture should not depend on the orientation of the picture. Invariant maps delete information since knowing *y* = *F*(*x*) does not allow to distinguish between *x* and *g* · *x*. If the invariant features required to solve a task are highly non-linear with respect to the inputs, then we might want to first transform the inputs before extracting any invariant information. And here we need to be careful, because if *H* is any map while *F* is invariant, it will not be true in general that *F*(*H*(*x*)) is invariant. On the other hand, we will see that if *H* is equivariant, then *F*(*H*(*x*)) will indeed be invariant. Let us now define equivariance: if *G* is a group acting on both spaces *X* and *Y*, and *H* : *X* → *Y* is a map between these spaces, then *H* is said to be equivariant if for any *g* ∈ *G* and any *x* ∈ *X*, we have *H*(*g* · *x*) = *g* · *H*(*x*). In words, it does not matter in which order we apply the group transformation and the map *H*. We can now verify our earlier claim: if *H* is equivariant and *F* is invariant, then *F*(*H*(*g* · *x*)) = *F*(*g* · *H*(*x*)) = *F*(*H*(*x*)), and *F* ◦ *H* is indeed invariant. As we will see later, this recipe of stacking equivariant maps followed by an invariant map, as shown in Figure 1B, is a commonly used recipe in ML (Bronstein et al., 2021).

So far we have considered discrete symmetries. However, many of the symmetries encountered in the real world are continuous. A group of symmetries is said to be continuous if there exist continuous paths between symmetries. For example, in the group of 2*D* rotations, we can create paths by smoothly varying the angle of the rotations. On the other hand, if we only allow rotations by multiple of 90°, then it is not possible to move smoothly from a rotation by 180° to a rotation by 270°. In that case, the group is said to be discrete.^5^ A simple approach to handle continuous symmetries used in practice in ML is to fall back to the discrete case by approximating the full group of continuous symmetries by a subgroup of discrete ones. For example, the group of rotations of the 2D plane can be approximated by only considering rotations by $\frac{360{}^{\circ}}{N}$, although this can become computationally expensive for very large groups (Finzi et al., 2020). While other approaches that truly handle a full group of continuous symmetries do exist (Rezende et al., 2019, 2020; Huang et al., 2020; Köhler et al., 2020; Pfau et al., 2020a; Cohen et al., 2021; Katsman et al., 2021; Papamakarios et al., 2021; Rezende and Racanière, 2021), we will concentrate on discrete symmetries in this paper for simplicity.

## 3. Implementation and Utility of Symmetries in ML

Although not always explicitly acknowledged, symmetries have been at the core of some of the most successful deep neural network architectures. For example, convolutional layers (CNNs) (LeCun and Bengio, 1995) responsible for the success of the deep classifiers that are able to outperform humans in their ability to categorize objects in images (Hu et al., 2018; Dai et al., 2021) are equivariant to translation symmetries characteristic of image classification tasks, while graph neural networks (GNNs) (Battaglia et al., 2018) and attention blocks commonly used in transformer architectures (Vaswani et al., 2017) are equivariant to the full group of permutations. While there are several reasons, including optimization considerations, why these architectural choices have been so successful compared to MLPs (Rosenblatt, 1958)—the original neural networks, one of the reasons is that these architectures reflect the prevalent symmetry groups of their respective data domains, while the linear layers used in MLPs are not compatible with any particular symmetry (Haykin, 1994), despite being theoretically proven universal function approximators (Cybenko, 1989; Hornik et al., 1989). Architectures like CNNs and GNNs reflect single type of symmetries (translations and permutations, respectively), but active research is also looking into building techniques to incorporate larger groups of symmetries into neural networks (Anselmi et al., 2013; Gens and Domingos, 2014; Cohen and Welling, 2016; Cohen et al., 2018).

**Figure 4:**
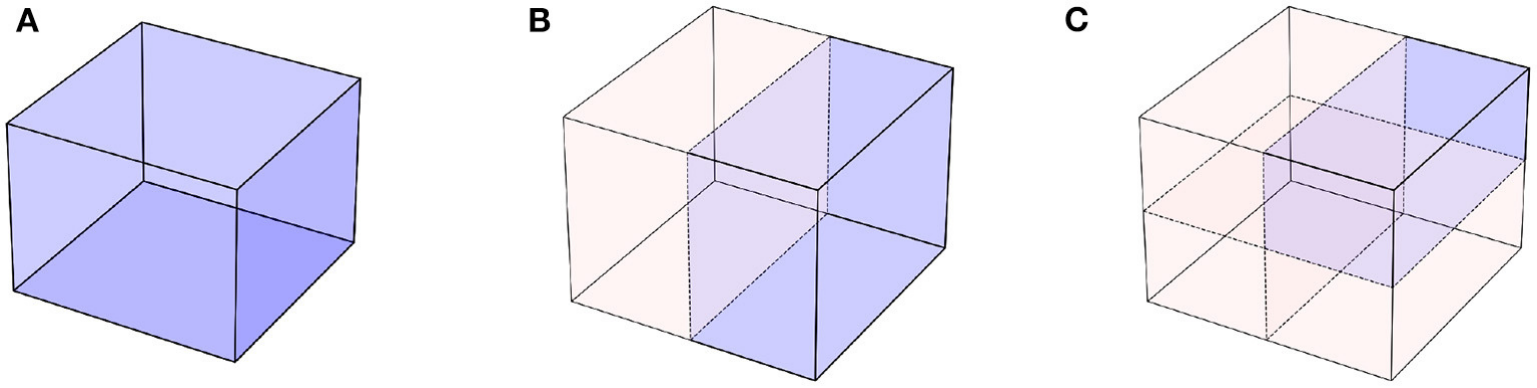
Symmetries let us reduce the volume of the domain on which our models need to learn. **(A)** The original problem domain. **(B)** With one symmetry, a reflection along a plane, we can half the domain on which we need to learn. **(C)** Further symmetries keep on reducing the volume of domain problem.

One of the main reasons why incorporating symmetries into neural networks helps is due to improvements in data efficiency. Indeed, incorporating symmetries can reduce the volume of the problem space, as illustrated in Figure 4. If we assume that the data processed by our model are points in a 3*D* cube (Figure 4A), when symmetries can be exploited, the models only need to work with a subset of the cube (Figures 4B,C), which reduces the volume of the input space. Provided the model respects symmetries by construction, learning on this reduced space is enough to learn on the entire cube. This naturally also leads to improvements in generalization and transfer, since new points outside of the training data distribution that can be obtained by applying the known symmetries to the observed data will be automatically recognizable. This principle has been exploited in scientific applications of ML, such as free energy estimation (Wirnsberger et al., 2020), protein folding (Fuchs et al., 2020; Baek et al., 2021), or quantum chemistry (Pfau et al., 2020b; Batzner et al., 2021).

An alternative to building symmetries into the model, is to use data-augmentation and let the model learn the symmetries. This is achieved by augmenting the training dataset (for example images) with the relevant transformations of this data (for example, all rotations and reflections of these images). This principle has been used as a source of augmentations for self-supervised contrastive learning approaches (Chen et al., 2020; Grill et al., 2020). While these approaches have been shown to be very effective in improving data efficiency on image classification tasks, other research has shown that learning symmetries from data augmentations is usually less effective than building them into the model architecture (Cohen and Welling, 2016; Qi et al., 2017; Veeling et al., 2018; Rezende et al., 2019; Köhler et al., 2020; Satorras et al., 2021).

An alternative to hard wiring inductive biases into the network architecture is to instead adjust the model's learning objective to make sure that its representations are equivariant to certain symmetries. This can be done implicitly by adding (unsupervised) regularizers to the main learning objective (Bellemare et al., 2017; Jaderberg et al., 2017), or explicitly by deciding on what a “good” representation should look like and directly optimizing for those properties. One example of the latter line of research is the work on disentangled^6^ representation learning (Bengio, 2009; Bengio et al., 2013) (also see related ideas in Schmidhuber, 1992; Hyvärinen, 1999). While originally proposed as an intuitive framework that suggested that the world can be described using a small number of independent generative factors, and the role of representation learning is to discover what these are and represent each generative factor in a separate representational dimension (Bengio et al., 2013), disentangling has recently been re-defined through a formal connection to symmetries (Higgins et al., 2019). In this view, a vector representation is seen as disentangled with respect to a particular decomposition of a symmetry group into a product of subgroups, if it can be decomposed into independent subspaces where each subspace is affected by the action of a single subgroup, and the actions of all the other subgroups leave the subspace unaffected.

**Figure 5:**
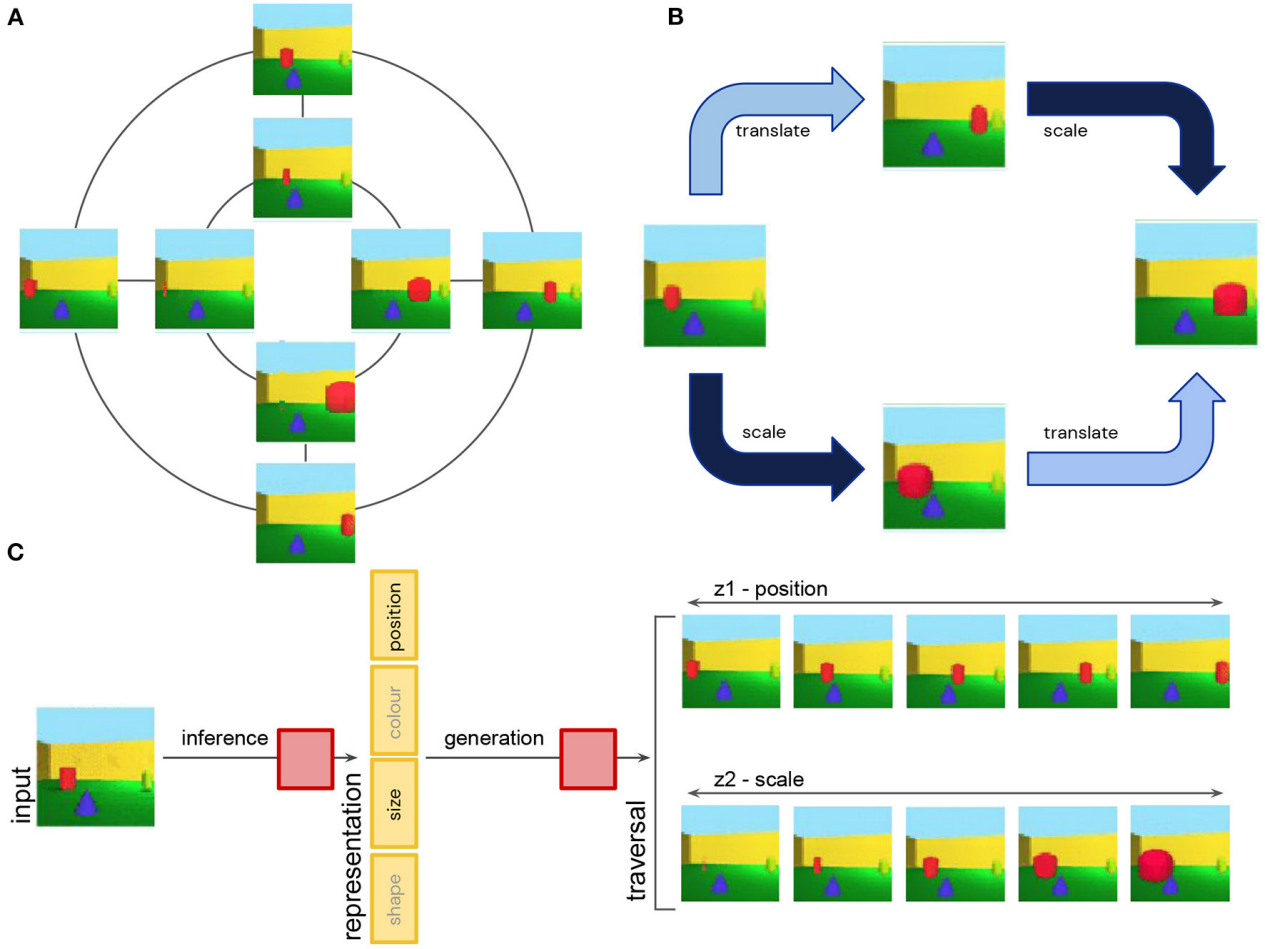
**(A)** Simplified schematic showing discrete approximation of continuous translation and scale symmetries of 3D objects. Translations are applied along the inner and outer circles. Scale transformations are applied along straight lines. **(B)** Translation and scale transformation commute with each other. They can be applied in permuted order without affecting the final state. **(C)** Disentangling neural networks learn to infer a representation of an image that is a concatenation of independent subspaces, each one being (approximately) equivariant to a single symmetry transformation. The model uses inference to obtain a low-dimensional representation of an image, and generation to reconstruct the original image from the representation. Two example latent traversals demonstrate the effect on the image reconstruction of smoothly varying the value of the position and size subspaces.

To understand this definition better, let's consider a concrete example of an object classification task (Figure 5A). Transformations like changes in the position or size of an object are symmetry transformations that keep the object identity invariant. These transformations also commute with each other, since they can be applied in random order without affecting the final state of the world (Figure 5B). This implies that the symmetry group used to describe the natural transformations in this world can be decomposed into a product of separate subgroups, including one subgroup that affects the position of an object, and another one affecting its size.

Assuming that the symmetry transformations act on a set of hypothetical ground truth abstract states of our world, and the disentangling model observes high-dimensional image renderings of such states, in which all the information about object identity, size and position among other factors is entangled, the goal of disentangled representation learning is to infer a representation which is decomposed into independent subspaces, where each subspace is affected only by a single subgroup of our original group of symmetry transformations. In other words, the vector space of such a representation would be a concatenation of independent subspaces, such that, for example, a change in size only affects the “size subspace,” but not the “position subspace” or any other subspace (Figure 5C). This definition of disentangled representations is very general—it does not assume any particular dimensionality or basis for each subspace. The changes along each of the subspaces in the representation may also be implemented by an arbitrary, potentially non-linear mapping, although if this mapping is linear, it can provide additional nice properties to the representation (Higgins et al., 2018 call such a representation a *linear disentangled representations*), since it means that the task relevant information (e.g., the “stable cores” of color or position attributes of the object) can be read out using linear decoders, and “nuisance” information can be easily ignored using a linear projection.

While the early approaches to disentangled representation learning (including related ideas from nonlinear dimensionality reduction literature, e.g., Hyvärinen, 1999; Hyvärinen and Pajunen, 1999; Tenenbaum et al., 2000; Belkin and Niyogi, 2001; Coifman and Lafon, 2006) either struggled to scale (Tenenbaum et al., 2000; Desjardins et al., 2012; Tang et al., 2013; Cohen and Welling, 2014, 2015) or relied on a form of supervision (Hinton et al., 2011; Reed et al., 2014; Zhu et al., 2014; Cheung et al., 2015; Goroshin et al., 2015; Kulkarni et al., 2015; Yang et al., 2015; Karaletsos et al., 2016; Whitney et al., 2016), most of the modern methods for successful unsupervised disentangling (Higgins et al., 2017a; Achille et al., 2018; Chen et al., 2018; Dupont, 2018; Kim and Mnih, 2018; Kumar et al., 2018; Ridgeway and Mozer, 2018; Ansari and Soh, 2019; Caselles-Dupré et al., 2019; Detlefsen and Hauberg, 2019; Dezfouli et al., 2019; Esmaeili et al., 2019; Lorenz et al., 2019; Mathieu et al., 2019; Ramesh et al., 2019; Lee et al., 2020; Quessard et al., 2020) are based on the Variational AutoEncoder (VAE) architecture (Kingma and Welling, 2014; Rezende et al., 2014)—a generative network that learns by predicting its own inputs. The base VAE framework learns a compressed representation that maximizes the marginal likelihood of the data and are related to the idea of “mean field approximation” from physics. In this framework no explicit desiderata are made about the representational form—as long as the distribution of the learnt data representation is close to the chosen prior (which often consists of independent unit Gaussians), it is considered to be acceptable. Disentangling VAEs, on the other hand, aim to learn a representation of a very particular form—it has to decompose into independent subspaces, each one reflecting the action of a single symmetry transformation. Disentangling VAEs typically work by adjusting the VAE learning objective to restrict the capacity of the representational bottleneck. This is usually done by encouraging the representation to be as close to the isotropic unit Gaussian distribution as possible, hence also encouraging factorization. Although it has been proven that unsupervised disentangled representation learning in this setting should be theoretically impossible (Locatello et al., 2019), these approaches work in practice by exploiting the interactions of the implicit biases in the data and the learning dynamics (Burgess et al., 2018; Locatello et al., 2019; Mathieu et al., 2019; Rolinek et al., 2019). Since these approaches are not optimizing for symmetry-based disentanglement directly, they are not principled and struggle to scale. However, they have been shown to learn an approximate symmetry-based disentangled representation (for example they often lose the cyclical aspect of the underlying symmetry) that still preserves much of the group structure (e.g., the commutativity of the symmetries) and hence serves as a useful tool for both understanding the benefits of symmetry-based representations in ML models, and as a computational model for studying representations in the brain (Soulos and Isik, 2020; Higgins et al., 2021a). In the meantime, new promising approaches to more scalable and/or principled disentanglement are starting to appear in the ML literature (Besserve et al., 2020; Pfau et al., 2020a; Higgins et al., 2021b; Wang et al., 2021).

In order to generalize learnt skills to new situations, it is helpful to base learning only on the smallest relevant subset of sensory variables, while ignoring everything else (Canas and Jones, 2010; Jones and Canas, 2010; Bengio et al., 2013; Niv et al., 2015; Leong et al., 2017; Niv, 2019). Symmetry-based representations make such attentional attenuation very easy, since meaningful sensory variables get separated into independent representational subspaces, as was demonstrated in a number of ML papers (Higgins et al., 2017b; Locatello et al., 2020). Following the reasoning described earlier, disentangled representations have also been shown to help with data efficiency when learning new tasks (Locatello et al., 2020; Wulfmeier et al., 2021). Finally, disentangled representations have also been shown to be a useful source of intrinsically motivated transferable skill learning. By learning how to control their own disentangled subspaces (e.g., how to control the position of an object), it has been shown that RL agents with disentangled representations could discover generally useful skills that could be readily re-used for solving new tasks (e.g., how to stack objects) in a more data efficient manner (Achille et al., 2018; Laversanne-Finot et al., 2018; Grimm et al., 2019; Wulfmeier et al., 2021).

## 4. Symmetries in Neuroscience

**Figure 6:**
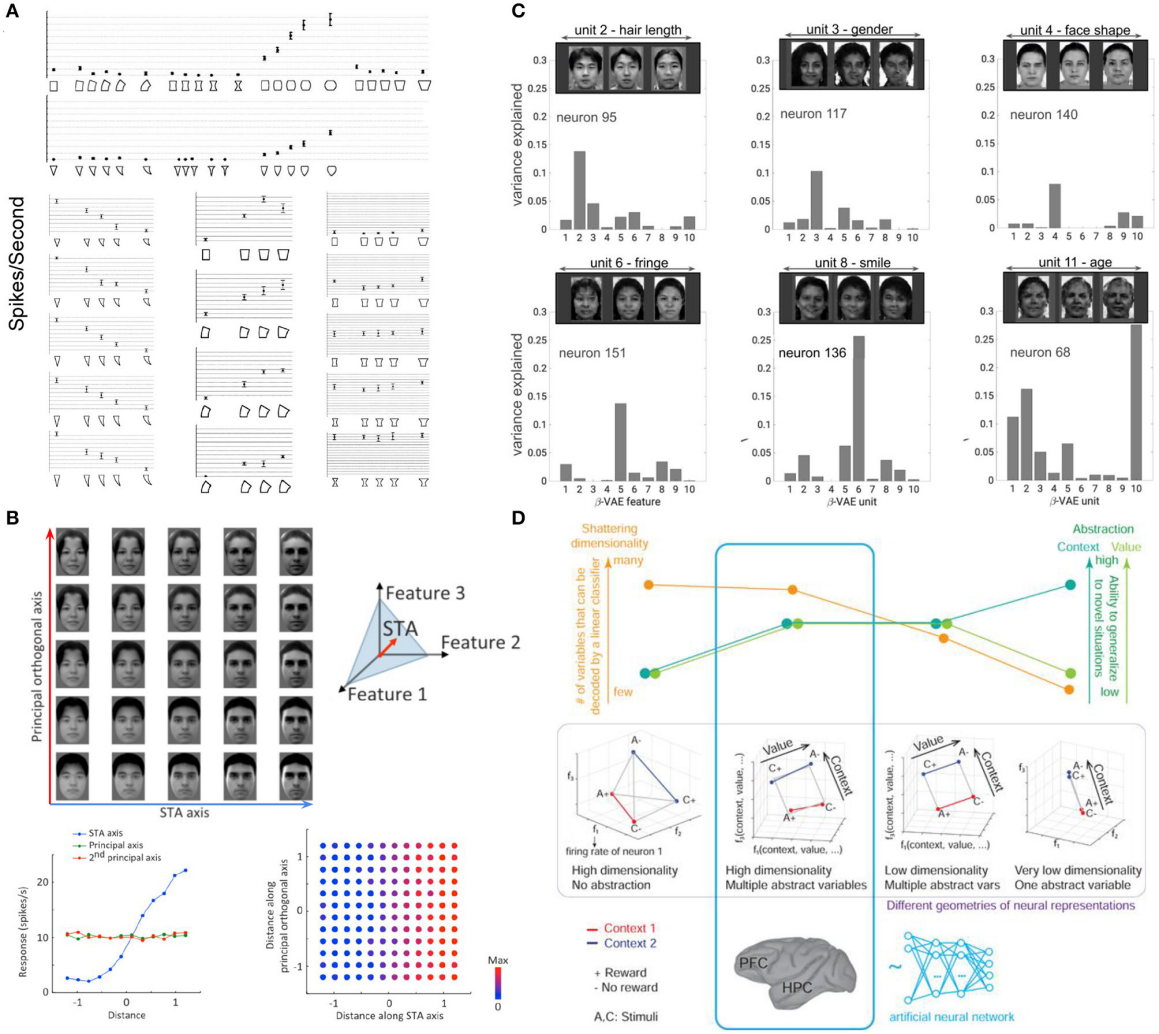
Examples of axis-based coding at the end of the ventral visual stream. **(A)** Single IT cell shows preference to a single transformation (change in positive curvature) regardless of the geometric shape (triangle or rectangle). Single IT cell responds to changes in curvature of the triangle while being invariant to changes in length, changes in tapering of the rectangle while being invariant to changes in curvature, and changes in negative curvature of the rectangle while being invariant to changes in tapering. Bars are standard errors in response to multiple stimulus presentations (14 on average). Adapted form Kayaert et al. (2005). **(B)** Single IT cells have ramped responses proportional to changes along their preferred axis of variation in the generative face space, and no changes in their responses to orthogonal directions in the face space. Adapted from Chang and Tsao (2017). **(C)** Single cells in the IT have strong one-to-one alignment to single subspaces discovered through disentangled representation learning. Adapted from Higgins et al. (2021a). **(D)** Different representation geometries have different trade-offs in terms of how much they support generalization as measured by the abstraction scores (green), and how expressive they are as measured by the shattering dimensionality score (orange). Representations in the prefrontal cortex (PFC) and hippocampus (HPS) of primates, as well as in the final layer of a neural network trained to solve multiple tasks in the reinforcement learning framework were found to exhibit disentangling-like geometry highlighted in blue that scores well on to both metrics. Adapted from Bernardi et al. (2020).

Although psychology and cognitive science picked up the mathematical framework of group theory to describe invariances and symmetry in vision a long time ago (Dodwell, 1983), this framework was not broadly adopted and progress in this direction quickly stalled (although see Liao et al., 2013; Leibo et al., 2017). However, circumstantial evidence from work investigating the geometry of neural representations suggests the possibility that the brain may be learning symmetry-based representations. For example, factorized representations of independent attributes, such as orientation and spatial frequency (Hubel and Wiesel, 1959; Mazer et al., 2002; Gáspár et al., 2019) or motion and direction tuning (Grunewald and Skoumbourdis, 2004) have long been known to exist at the start of the ventral visual stream in V1. Going further along the visual hierarchy, Kayaert et al. (2005) demonstrated that many of the primate IT neurons had monotonic tuning to the generative dimensions of toy visual stimuli, such as curvature, tapering or aspect ratio, known to be discriminated independently from each other by humans in psychophysical studies (Arguin and Saumier, 2000; Stankiewicz, 2002; de Beeck et al., 2003). In particular, they found that the firing of each neuron was modulated strongly by its preferred generative attribute but significantly less so by the other generative attributes (Figure 6A).

More recently, Chang and Tsao (2017) investigated the coding properties of single IT neurons in the primate face patches. By parameterizing the space of faces using a low-dimensional code, they were able to show that each neuron was sensitive to a specific axis in the space of faces spanned by as few as six generative dimensions on average, with different cells preferring different axes. Moreover, the recorded IT cells were found to be insensitive to changes in directions orthogonal to their preferred axis, suggesting a low-dimensional factorized representation reminiscent of disentangled representations from ML (Figure 6B). To directly test whether the two representations resembled each other, Higgins et al. (2021a) compared the responses of single cells in the IT face patches to disentangled latent units discovered by a model exposed to the same faces as the primates (Figure 6C). By measuring the alignment between the two manifolds, the authors were able to compare the two representational forms in a way that was sensitive to linear transformations (unlike the traditional measures of similarity used in the neuroscience literature, like explained variance Cadieu et al., 2007; Khaligh-Razavi and Kriegeskorte, 2014; Güçlü and van Gerven, 2015; Yamins and DiCarlo, 2016; Cadena et al., 2019 or Representational Similarity Analysis Kriegeskorte et al., 2008; Khaligh-Razavi and Kriegeskorte, 2014, which are invariant to linear transformations)—any rotation or shear of one manifold with respect to the other would result in reduced scores. The authors found that there was a strong one-to-one alignment between IT neurons and disentangled units to the point where the small number of disentangled dimensions discovered by the model were statistically equivalent to a similarly sized subset of real neurons, and the alignment was significantly stronger than that with supervised classifiers (which learn an invariant representation) or the generative model used in Chang and Tsao (2017). Furthermore, it was possible to visualize novel faces viewed by the primates from the decoded activity of just 12 neurons through their best matched disentangled units. This result established the first direct link between coding in single IT neurons and disentangled representations, suggesting that the brain may be learning representations that reflect the symmetries of the world. Other recent work showed that disentangled representations can also predict fMRI activation in the ventral visual stream (Soulos and Isik, 2020).

While many of the existing approaches to disentangled representation learning are generative models, thus fitting well within the predictive coding and free energy principle (Elias, 1955; Srinivasan et al., 1982; Rao and Ballard, 1999; Friston, 2010; Clark, 2013) hypotheses of brain function, an alternative biologically plausible way to learn disentangled representations was recently proposed by Johnston and Fusi (2021). The authors showed that disentangled representations can arise from learning to solve numerous diverse tasks in a supervised manner, which would be required to produce the complex behaviors that biological intelligence exhibits in the natural world. A similar result was also demonstrated by Bernardi et al. (2020), who looked into the geometry of neural representations for solving tasks in the RL framework in both primates and neural networks. They found that the final layer of an MLP trained through RL supervision to solve a number of tasks, as well as the dorsolateral prefrontal cortex, the anterior cingulate cortex and the hippocampus of primates exhibited disentangled-like qualities. Although the representations of the underlying task variables were rotated in the space of neural activation (unlike the axis aligned codes described in Higgins et al., 2021a), the underlying geometry was in line with what would be expected from disentangled representations (see also Minxha et al., 2020; Panichello and Buschman, 2021; Rodgers et al., 2021; She et al., 2021; Boyle et al., 2022 for further evidence of not axis-aligned disentangled-like representations in different brain areas of various species). The authors found that the degree to which such geometry was present correlated with the primates success on the tasks (no such correlation existed for the more traditional decoding methods that do not take the geometry of the representation into account), and that such representations supported both strong generalization (as measured by the abstraction scores) and high representational capacity (as measured by the shattering dimensionality scores) (Figure 6D).

**Figure 7:**
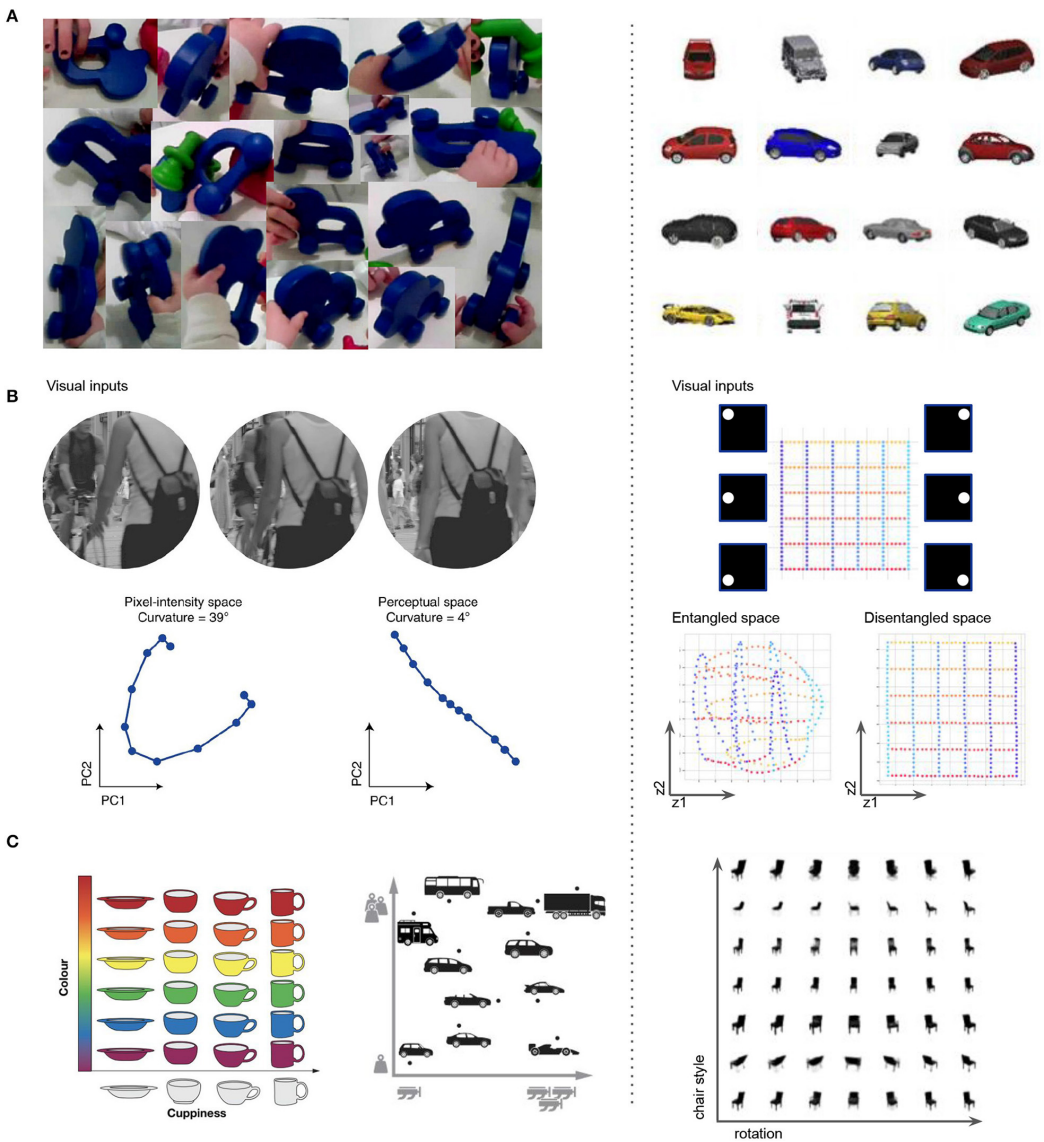
Similarities between various aspects of disentangled representation learning in ML (right column) and visual representation learning in the brain (left column). **(A)** The properties of the visual data obtained through a head camera from toddlers (Smith et al., 2018; Slone et al., 2019) is similar to the properties of the visual data that allows ML approaches to discover disentangled representations. The scenes are uncluttered, and contain many continuous transformations of few objects at a time. **(B)** Perceptual straightening of natural image trajectories observed in human vision (Hénaff et al., 2019) is similar to the “Euclidenization” of the latent space learnt by disentangled ML models. **(C)** Factorized representations that align with semantically meaningful attributes hypothesized to be important for further processing in the hippocampus (Behrens et al., 2018; Bellmund et al., 2018) resembles the factorized representations learnt by disentangled ML models.

Further validation of the biological plausibility of disentangled representation learning comes from comparing the data distribution that many modern ML approaches require for optimal disentangling to the early visual experiences of infants (Smith et al., 2018; Wood and Wood, 2018; Slone et al., 2019). It appears that the two are similar, with smooth transformations of single objects dominating both (Figure 7A). Disentangled representation also have properties that are believed to be true of the visual brain, such as “Euclideanization” or straightening of complex non-linear trajectories in the representation space compared to the input observation space (Hénaff et al., 2019) (Figure 7B), and factorization into semantically interpretable axes, such as color or shape of objects (Figure 7C), which are hypothesized to be important for more data efficient and generalizable learning (Behrens et al., 2018), and for supporting abstract reasoning (Bellmund et al., 2018). It is hypothesized that the same principles that allow biological intelligence to navigate the physical space using the place and grid cells may also support navigation in cognitive spaces of concepts, where concepts are seen as convex regions in a geometric space spanned by meaningful axes like engine power and car weight (Gärdenfors, 2004; Gardenfors, 2014; Balkenius and Gärdenfors, 2016). Learning disentangled representations that reflect the symmetry structure of the world could be a plausible mechanism for discovering such axes. Evidence from the ML literature has already demonstrated the utility of disentangled representations for basic visual concept learning, imagination, and abstract reasoning (Higgins et al., 2018; Steenbrugge et al., 2018; van Steenkiste et al., 2019; Locatello et al., 2020).

## 5. Discussion

The question of what makes a good representation has been historically central to both ML and neuroscience, and both disciplines have faced the same debate: whether the best representation to support intelligent behavior should be low-dimensional and interpretable or high-dimensional and multiplexed. While the former dominated both early neuroscience (Hubel and Wiesel, 1959; Barlow, 1972) and ML (early success of feature engineering), recent development of high-throughput recording methods in neuroscience (Yuste, 2015; Eichenbaum, 2018; Saxena and Cunningham, 2019) and the success of large black-box deep learning models in ML (Vaswani et al., 2017; Hu et al., 2018) have shifted the preference in both fields toward the latter. As a consequence, this led to deep classifiers emerging as the main computational models for the ventral visual stream (Yamins et al., 2014; Yamins and DiCarlo, 2016), and a belief that higher-level sensory representations that can support diverse tasks are too complex to interpret at a single neuron level. This pessimism was compounded by the fact that designing stimuli for discovering interpretable tuning in single cells at the end of the sensory processing pathways is hard. While it is easy to systematically vary stimulus identity, it is hard to know what the other generative attributes of complex natural stimuli may be, and hence to create stimuli that systematically vary along those dimensions. Furthermore, new representation comparison techniques between computational models and the brain became progressively population-based and insensitive to linear transformations (Kriegeskorte et al., 2008; Khaligh-Razavi and Kriegeskorte, 2014; Yamins and DiCarlo, 2016), thus further stalling progress toward gaining a more fine-grained understanding of the representational form utilized by the brain (Thompson et al., 2016; Higgins et al., 2021a). At the same time, it is becoming increasingly unlikely that high-dimensional, multiplexed, uninterpretable population-based representations like those learnt by deep classifiers are the answer to what makes a “good” representation to support general intelligence, since ML research has shown that models with such representations suffer from problems in terms of data efficiency, generalization, transfer, and robustness—all the properties that are characteristic of biological general intelligence. In this article, we have argued that representations which reflect the natural symmetry transformations of the world may be a plausible alternative. This is because both the nature of the tasks, and the evolutionary development of biological intelligence are constrained by physics, and physicists have been using symmetry transformations to discover and study the “joints” and the “stable cores” of the world for the last century. By studying symmetry transformations, physicists have been able to reconcile explanatory frameworks, systematically describe physical objects and even discover new ones. Representations that are equivariant to symmetry transformations are therefore likely to expose the relevant invariants of our world that are useful for solving natural tasks. From the information theory perspective, such representations can be viewed as the simplest (in the context of Solomonoff induction; Solomonoff, 1964) and the most informative representations of the input to support the most likely future tasks (MacKay, 1995, 2003; Wallace and Dowe, 1999; Hutter, 2004; Schmidhuber, 2010).

We have introduced the basic mathematical language for describing symmetries, and discussed evidence from ML literature that demonstrates the power of symmetry-based representations in bringing better data efficiency, generalization, and transfer when included into ML systems. Furthermore, emerging evidence from the neuroscience community suggests that sensory representations in the brain may also be symmetry-based. We hope that our review will give the neuroscience community the necessary motivation and tools to look further into how symmetries can explain representation learning in the brain, and to consider them as an important general framework that determines the structure of the universe, constrains the nature of natural tasks and consequently shapes both biological and artificial intelligence.

## Author Contributions

IH and SR contributed to writing the review. DR contributed comments, discussions, and pointers that shaped the paper. All authors contributed to the article and approved the submitted version.

## Conflict of Interest

IH, SR, and DR were employed by DeepMind.

## Publisher's Note

All claims expressed in this article are solely those of the authors and do not necessarily represent those of their affiliated organizations, or those of the publisher, the editors and the reviewers. Any product that may be evaluated in this article, or claim that may be made by its manufacturer, is not guaranteed or endorsed by the publisher.

## Footnotes

^1^These are also referred to as “grandmother cells” in the literature.

^2^This kind of action is distinct from the action in physics; here, it just refers to the action of an operator.

^3^To be precise, we are defining here a *left* action.

^4^The term *Abelian* is also used in the literature.

^5^Some groups will have both continuous and discrete aspects. For example, the group of all invertible matrices of a given size has a clear continuous aspect, but it also has a discrete aspect as we cannot move continuously from a matrix with positive determinant to a matrix with negative determinant without hitting a matrix with determinant 0.

^6^Although the term “disentanglement” and its opposite “entanglement” are also used in quantum mechanics (QM), and indeed the term “entanglement” refers to a mixing of factors in both ML (through any diffeomorphism) and QM (through a linear combinations), there is no deeper connection between the two.

## References

[B1] Achille A. Eccles T. Matthey L. Burgess C. P. Watters N. Lerchner A.. (2018). Life-long disentangled representation learning with cross-domain latent homologies, in Advances in Neural Information Processing Systems (NeurIPS) (Montreal, QC).

[B2] Agostini A. Celaya E. (2009). Exploiting domain symmetries in reinforcement learning with continuous state and action spaces, in 2009 International Conference on Machine Learning and Applications (Montreal, QC), 331–336. 10.1109/ICMLA.2009.41

[B3] Anand A. Grover A. Singla P. (2016). Contextual symmetries in probabilistic graphical models. arXiv preprint: arXiv:1606.09594. 10.48550/arXiv.1606.09594

[B4] Ansari A. F. Soh H. (2019). Hyperprior induced unsupervised disentanglement of latent representations, in Proceedings of the Thirty-Third AAAI Conference on Artificial Intelligence (AAAI) (Honolulu). 10.1609/aaai.v33i01.33013175

[B5] Anselmi F. Leibo J. Z. Rosasco L. Mutch J. Tacchetti A. Poggio T. (2013). Unsupervised learning of invariant representations in hierarchical architectures. arXiv preprint: arXiv:1311.4158. 10.48550/arXiv.1311.415824877728

[B6] Arguin M. Saumier D. (2000). Conjunction and linear non-separability effects in visual shape encoding. Vis. Res. 40, 3099–3115. 10.1016/S0042-6989(00)00155-3 10996614

[B7] Baek M. DiMaio F. Anishchenko I. Dauparas J. Ovchinnikov S. Lee G. R.. (2021). Accurate prediction of protein structures and interactions using a three-track neural network. Science 373, 871–876. 10.1126/science.abj8754 34282049 PMC7612213

[B8] Balkenius C. Gärdenfors P. (2016). Spaces in the brain: from neurons to meanings. Front. Psychol. 7:1820. 10.3389/fpsyg.2016.01820 27920740 PMC5118439

[B9] Barlow H. B. (1972). Single units and sensation: a neuron doctrine for perceptual psychology? Perception 1, 371–394. 10.1068/p010371 4377168

[B10] Battaglia P. W. Hamrick J. B. Bapst V. Sanchez-Gonzalez A. Zambaldi V. Malinowski M.. (2018). Relational inductive biases, deep learning, and graph networks. arXiv preprint: arXiv:1806.01261. 10.48550/arXiv.1806.0126135156011

[B11] Batzner S. Musaelian A. Sun L. Geiger M. Mailoa J. P. Kornbluth M.. (2021). SE(3)-equivariant graph neural networks for data-efficient and accurate interatomic potentials. arXiv preprint: arXiv:2101.03164. 10.21203/rs.3.rs-244137/v1 PMC9068614 35508450

[B12] Behrens T. E. Muller T. H. Whittington J. C. Mark S. Baram A. B. Stachenfeld K. L.. (2018). What is a cognitive map? organizing knowledge for flexible behavior. Neuron 100, 490–509. 10.1016/j.neuron.2018.10.002 30359611

[B13] Belkin M. Niyogi P. (2001). Laplacian eigenmaps and spectral techniques for embedding and clustering, in Advances in Neural Information Processing Systems (Vancouver, BC), 585–591.

[B14] Bellemare M. G. Dabney W. Munos R. (2017). A distributional perspective on reinforcement learning, in International Conference on Machine Learning (Sydney), 449–458.

[B15] Bellmund J. L. S. Gärdenfors P. Moser E. I. Doeller C. F. (2018). Navigating cognition: spatial codes for human thinking. Science 362:6415. 10.1126/science.aat6766 30409861

[B16] Bengio Y. (2009). Learning deep architectures for AI. Found. Trends Mach. Learn. 2, 1–127. 10.1561/9781601982957

[B17] Bengio Y. (2012). Deep learning of representations for unsupervised and transfer learning, in Proceedings of ICML Workshop on Unsupervised and Transfer Learning, eds Guyon I. Dror G. Lemaire V. Taylor G. Silver D. (Washington, DC: PMLR), 17–36. Available online at: http://proceedings.mlr.press/v27/bengio12a/bengio12a.pdf

[B18] Bengio Y. Courville A. Vincent P. (2013). Representation learning: a review and new perspectives. IEEE Trans. Pattern Anal. Mach. Intell. 35, 1798–1828. 10.1109/TPAMI.2013.50 23787338

[B19] Bernardi S. Benna M. K. Rigotti M. Munuera J. Fusi S. Salzman C. D. (2020). The geometry of abstraction in the hippocampus and prefrontal cortex. Cell 183, 954–967. 10.1016/j.cell.2020.09.031 33058757 PMC8451959

[B20] Besserve M. Mehrjou A. Sun R. Scholkopf B. (2020). Counterfactuals uncover the modular structure of deep generative models, in International Conference on Learning Representations. Available online at: https://openreview.net/forum?id=SJxDDpEKvH

[B21] Boyle L. Posani L. Irfan S. Siegelbaum S. A. Fusi S. (2022). The geometry of hippocampal CA2 representations enables abstract coding of social familiarity and identity. bioRxiv [Preprint]. 10.1101/2022.01.24.477361

[B22] Bronstein M. M. Bruna J. Cohen T. Veličković P. (2021). Geometric deep learning: Grids, groups, graphs, geodesics, and gauges. arXiv preprint: arXiv:2104.13478. 10.48550/arXiv.2104.13478

[B23] Burgess C. P. Higgins I. Pal A. Matthey L. Watters N. Desjardins G.. (2018). Understanding disentangling in β-VAE. arXiv preprint: arXiv:1804.03599. 10.48550/arXiv.1804.03599

[B24] Cadena S. A. Denfield G. H. Walker E. Y. Gatys L. A. Tolias A. S. Bethge M.. (2019). Deep convolutional models improve predictions of macaque v1 responses to natural images. PLoS Comput. Biol. 15:e1006897. 10.1371/journal.pcbi.1006897 31013278 PMC6499433

[B25] Cadieu C. Kouh M. Pasupathy A. Connor C. E. Riesenhuber M. Poggio T. (2007). A model of v4 shape selectivity and invariance. J. Neurophysiol. 98, 1733–1750. 10.1152/jn.01265.2006 17596412

[B26] Canas F. Jones M. (2010). Attention and reinforcement learning: constructing representations from indirect feedback, in Proceedings of the Annual Meeting of the Cognitive Science Society, Vol. 32 (Portland).

[B27] Caselles-Dupré H. Garcia-Ortiz M. Filliat D. (2019). Symmetry-based disentangled representation learning requires interaction with environments, in Advances in Neural Information Processing Systems (NeurIPS) (Vancouver, BC).

[B28] Chang L. Tsao D. Y. (2017). The code for facial identity in the primate brain. Cell 169:1013-1028.e14. 10.1016/j.cell.2017.05.011 28575666 PMC8088389

[B29] Chen T. Kornblith S. Norouzi M. Hinton G. (2020). A simple framework for contrastive learning of visual representations, in International Conference on Machine Learning (Vienna), 1597–1607.

[B30] Chen T. Q. Li X. Grosse R. Duvenaud D. (2018). Isolating sources of disentanglement in variational autoencoders, in Advances in Neural Information Processing Systems (NeurIPS) (Montreal, QC). 10.1007/978-3-030-04167-0

[B31] Cheung B. Levezey J. A. Bansal A. K. Olshausen B. A. (2015). Discovering hidden factors of variation in deep networks, in Proceedings of the International Conference on Learning Representations, Workshop Track (San Diego, CA).

[B32] Clark A. (2013). Whatever next? Predictive brains, situated agents and the future of cognitive science. Behav. Brain Sci. 36, 181–204. 10.1017/S0140525X12000477 23663408

[B33] Cobbe K. Klimov O. Hesse C. Kim T. Schulman J. (2019). Quantifying generalization in reinforcement learning, in International Conference on Machine Learning (Long Beach, CA), 1282–1289.

[B34] Cohen S. Amos B. Lipman Y. (2021). Riemannian convex potential maps, in International Conference on Machine Learning (PMLR), 2028–2038.

[B35] Cohen T. Welling M. (2014). Learning the irreducible representations of commutative lie groups, in International Conference on Machine Learning (PMLR), 1755–1763.

[B36] Cohen T. Welling M. (2015). Transformation properties of learned visual representations, in ICLR (San Diego, CA).

[B37] Cohen T. Welling M. (2016). Group equivariant convolutional networks, in International Conference on Machine Learning, eds Balcan M. F. Weinberger K. Q. (New York, NY: PMLR), 2990–2999. Available online at: http://proceedings.mlr.press/v48/cohenc16.pdf

[B38] Cohen T. S. Geiger M. Kohler J. Welling M. (2018). Spherical CNNs, in International Conference on Learning Representations. Available online at: https://openreview.net/forum?id=Hkbd5xZRb

[B39] Coifman R. R. Lafon S. (2006). Diffusion maps. Appl. Comput. Harmon. Anal. 21, 5–30. 10.1016/j.acha.2006.04.006

[B40] Cybenko G. (1989). Approximation by superpositions of a sigmoidal function. Math. Control Signals Syst. 2, 303–314. 10.1007/BF02551274

[B41] Dabney W. Kurth-Nelson Z. Uchida N. Starkweather C. K. Hassabis D. Munos R.. (2020). A distributional code for value in dopamine-based reinforcement learning. Nature 577, 671–675. 10.1038/s41586-019-1924-6 31942076 PMC7476215

[B42] Dai Z. Liu H. Le Q. Tan M. (2021). Coatnet: Marrying convolution and attention for all data sizes, in Advances in Neural Information Processing Systems.

[B43] Dalal N. Triggs B. (2005). Histograms of oriented gradients for human detection, in IEEE Computer Society Conference on Computer Vision and Pattern Recognition, 2005, CVPR 2005, Vol. 1 (Boston, MA), 886–893. 10.1109/CVPR.2005.177

[B44] de Beeck H. O. Wagemans J. Vogels R. (2003). The effect of category learning on the representation of shape: dimensions can be biased but not differentiated. J. Exp. Psychol. 132:491. 10.1037/0096-3445.132.4.491 14640844

[B45] Desjardins G. Courville A. Bengio Y. (2012). Disentangling factors of variation *via* generative entangling. arXiv:1210.5474. 10.48550/arXiv.1210.5474

[B46] Detlefsen N. S. Hauberg S. (2019). Explicit disentanglement of appearance and perspective in generative models, in Advances in Neural Information Processing Systems (NeurIPS) (Vancouver, BC).

[B47] Dezfouli A. Ashtiani H. Ghattas O. Nock R. Dayan P. Ong C. S. (2019). Disentangled behavioral representations, in Advances in Neural Information Processing Systems (NeurIPS) (Vancouver, BC). 10.1101/658252

[B48] DiCarlo J. Zoccolan D. Rust N. (2012). How does the brain solve visual object recognition? Neuron 73, 415–434. 10.1016/j.neuron.2012.01.010 22325196 PMC3306444

[B49] DiCarlo J. J. Cox D. D. (2007). Untangling invariant object recognition. Trends Cogn. Sci. 11, :333–341. 10.1016/j.tics.2007.06.010 17631409

[B50] Dodwell P. C. (1983). The lie transformation group model of visual perception. Percept. Psychophys. 34, 1–16. 10.3758/BF03205890 6634353

[B51] Dummit D. S. Foote R. M. (1991). Abstract Algebra, Vol. 1999. Englewood Cliffs, NJ: Prentice Hall.

[B52] Dupont E. (2018). Learning disentangled joint continuous and discrete representations, in Advances in Neural Information Processing Systems (NeurIPS) (Montreal, QC).

[B53] Eichenbaum H. (2018). Barlow versus Hebb: when is it time to abandon the notion of feature detectors and adopt the cell assembly as the unit of cognition? Neurosci. Lett. 680, 88–93. 10.1016/j.neulet.2017.04.006 28389238 PMC5628090

[B54] Elias P. (1955). Predictive coding-i. IRE Trans. Inform. Theory 1, 16–24. 10.1109/TIT.1955.1055126

[B55] Esmaeili B. Wu H. Jain S. Bozkurt A. Siddharth N. Paige B.. (2019). Structured disentangled representations, in Proceedings of the 22nd International Conference on Artificial Intelligence and Statistics (AISTATS) (Okinawa).

[B56] Espeholt L. Soyer H. Munos R. Simonyan K. Mnih V. Ward T.. (2018). Impala: Scalable distributed deep-rl with importance weighted actor-learner architectures, in International Conference on Machine Learning (PMLR), 1407–1416.

[B57] Finzi M. Stanton S. Izmailov P. Wilson A. G. (2020). Generalizing convolutional neural networks for equivariance to lie groups on arbitrary continuous data, in International Conference on Machine Learning (Vienna), 3165–3176.

[B58] Friston K. (2010). The free-energy principle: a unified brain theory? Nat. Rev. Neurosci. 11, 127–138. 10.1038/nrn2787 20068583

[B59] Fuchs F. Worrall D. Fischer V. Welling M. (2020). Se (3)-transformers: 3d roto-translation equivariant attention networks, in Advances in Neural Information Processing Systems, 1970–1981.

[B60] Fukushima K. (1980). A self-organizing neural network model for a mechanism of pattern recognition unaffected by shift in position. Biol. Cybern. 36, 193–202. 10.1007/BF00344251 7370364

[B61] Gärdenfors P. (2004). Conceptual Spaces: The Geometry of Thought. Cambridge, MA: MIT Press.

[B62] Gardenfors P. (2014). The Geometry of Meaning: Semantics Based on Conceptual Spaces. Cambridge, MA: MIT Press. 10.7551/mitpress/9629.001.0001

[B63] Garnelo M. Arulkumaran K. Shanahan M. (2016). Towards deep symbolic reinforcement learning. arXiv preprint: arXiv:1609.05518. 10.48550/arXiv.1609.05518

[B64] Gáspár M. E. Polack P.-O. Golshani P. Lengyel M. Orbán G. (2019). Representational untangling by the firing rate nonlinearity in V1 simple cells. eLife 8:43625. 10.7554/eLife.43625 31502537 PMC6739864

[B65] Gens R. Domingos P. M. (2014). Deep symmetry networks, in NIPS (Montreal, QC).

[B66] Goroshin R. Mathieu M. LeCun Y. (2015). Learning to linearize under uncertainty, in NIPS (Montreal, QC).

[B67] Grill J. -B. Strub F. Altche F. Tallec C. Richemond P. Buchatskaya E.. (2020). Bootstrap your own latent-a new approach to self-supervised learning, in Advances in Neural Information Processing Systems, 33, 21271–21284.

[B68] Grimm C. Higgins I. Barreto A. Teplyashin D. Wulfmeier M. Hertweck T.. (2019). Disentangled cumulants help successor representations transfer to new tasks. arXiv preprint: arXiv:1911.10866. 10.48550/arXiv.1911.10866

[B69] Grunewald A. Skoumbourdis E. K. (2004). The integration of multiple stimulus features by v1 neurons. J. Neurosci. 24, 9185–9194. 10.1523/JNEUROSCI.1884-04.2004 15483137 PMC6730054

[B70] Güçlü U. van Gerven M. A. (2015). Deep neural networks reveal a gradient in the complexity of neural representations across the ventral stream. J. Neurosci. 35, 10005–10014. 10.1523/JNEUROSCI.5023-14.2015 26157000 PMC6605414

[B71] Hassabis D. Kumaran D. Summerfield C. Botvinick M. (2017). Neuroscience-inspired artificial intelligence. Neuron 95, 245–258. 10.1016/j.neuron.2017.06.011 28728020

[B72] Haykin S. (1994). Neural Networks: A Comprehensive Foundation. New York, NY: Prentice Hall.

[B73] Hénaff O. J. Goris R. L. Simoncelli E. P. (2019). Perceptual straightening of natural videos. Nat. Neurosci. 22, 984–991. 10.1038/s41593-019-0377-4 31036946

[B74] Higgins I. Amos D. Pfau D. Racaniere S. Matthey L. Rezende D.. (2019). Towards a definition of disentangled representations, in Theoretical Physics for Deep Learning Workshop, ICML (Long Beach, CA).

[B75] Higgins I. Chang L. Langston V. Hassabis D. Summerfield C. Tsao D.. (2021a). Unsupervised deep learning identifies semantic disentanglement in single inferotemporal face patch neurons. Nat. Commun. 12:6456. 10.1038/s41467-021-26751-5 34753913 PMC8578601

[B76] Higgins I. Matthey L. Pal A. Burgess C. Glorot X. Botvinick M.. (2017a). β-vae: learning basic visual concepts with a constrained variational framework, in ICLR (Toulon).

[B77] Higgins I. Pal A. Rusu A. Matthey L. Burgess C. Pritzel A.. (2017b). DARLA: improving zero-shot transfer in reinforcement learning, in ICML (Sydney).

[B78] Higgins I. Sonnerat N. Matthey L. Pal A. Burgess C. Bosnjak M.. (2018). SCAN: Learning hierarchical compositional visual concepts, in ICLR (Vancouver).

[B79] Higgins I. Wirnsberger P. Jaegle A. Botev A. (2021b). Symetric: measuring the quality of learnt hamiltonian dynamics inferred from vision, in Thirty-Fifth Conference on Neural Information Processing Systems.

[B80] Hinton G. Krizhevsky A. Jaitly N. Tieleman T. Tang Y. (2012). Does the brain do inverse graphics?, in Brain and Cognitive Sciences Fall Colloquium, Vol. 2.

[B81] Hinton G. E. Krizhevsky A. Wang S. D. (2011). Transforming auto-encoders, in International Conference on Artificial Neural Networks, eds Honkela T. Duch W. Girolami M. Kaski S. (Berlin; Heidelberg: Springer), 44–51.

[B82] Hornik K. Stinchcombe M. White H. (1989). Multilayer feedforward networks are universal approximators. Neural Netw. 2, 359–366. 10.1016/0893-6080(89)90020-8

[B83] Hu J. Shen L. Sun G. (2018). Squeeze-and-excitation networks, in Proceedings of the IEEE Conference on Computer Vision and Pattern Recognition (IEEE), 7132–7141. 10.1109/CVPR.2018.00745

[B84] Huang C.-W. Chen R. T. Tsirigotis C. Courville A. (2020). Convex potential flows: universal probability distributions with optimal transport and convex optimization. arXiv preprint: arXiv:2012.05942. 10.48550/arXiv.2012.05942

[B85] Hubel D. H. Wiesel T. N. (1959). Receptive fields of single neurones in the cat's striate cortex. J. Physiol. 124, 574–591. 10.1113/jphysiol.1959.sp006308 14403679 PMC1363130

[B86] Hutter M. (2004). Universal Artificial Intelligence: Sequential Decisions Based on Algorithmic Probability. Berlin: Springer Science & Business Media.

[B87] Hyvärinen A. (1999). Survey on Independent Component Analysis (Citeseer). Available online at: https://www.cs.helsinki.fi/u/ahyvarin/papers/NCS99.pdf

[B88] Hyvärinen A. Pajunen P. (1999). nonlinear independent component analysis: existence and uniqueness results. Neural Netw. 12, 429–439. 10.1016/S0893-6080(98)00140-3 12662686

[B89] Jaderberg M. Mnih V. Czarnecki W. M. Schaul T. Leibo J. Z. Silver D.. (2017). Reinforcement learning with unsupervised auxiliary tasks, in ICLR (Toulon).

[B90] Johnston W. J. Fusi S. (2021). Abstract representations emerge naturally in neural networks trained to perform multiple tasks. bioRxiv. 10.1101/2021.10.20.465187 PMC9950464 36823136

[B91] Jones M. Canas F. (2010). Integrating reinforcement learning with models of representation learning, in Proceedings of the Annual Meeting of the Cognitive Science Society, Vol. 32 (Portland).

[B92] Kansky K. Silver T. Mély D. A. Eldawy M. Lázaro-Gredilla M. Lou X.. (2017). Schema networks: Zero-shot transfer with a generative causal model of intuitive physics, in International Conference on Machine Learning (Sydney), 1809–1818.

[B93] Karaletsos T. Belongie S. Rätsch G. (2016). Bayesian representation learning with oracle constraints, in ICLR (san juan).

[B94] Katsman I. Lou A. Lim D. Jiang Q. Lim S.-N. De Sa C. (2021). Equivariant manifold flows, in ICML Workshop on Invertible Neural Networks, Normalizing Flows, and Explicit Likelihood Models.

[B95] Kayaert G. Biederman I. Op de Beeck H. P. Vogels R. (2005). Tuning for shape dimensions in macaque inferior temporal cortex. Eur. J. Neurosci. 22, 212–224. 10.1111/j.1460-9568.2005.04202.x 16029211

[B96] Khaligh-Razavi S. Kriegeskorte N. (2014). Deep supervised, but not unsupervised, models may explain IT cortical representation. PLoS Comput. Biol. 10:e1003915. 10.1371/journal.pcbi.1003915 25375136 PMC4222664

[B97] Kim H. Mnih A. (2018). Disentangling by factorizing, in Proceedings of the Sixth Annual International Conference on Learning Representations (ICLR) (Vancouver, BC).

[B98] Kingma D. P. Welling M. (2014). Auto-encoding variational Bayes, in ICLR (Banff, CN).

[B99] Kirsch L. Flennerhag S. van Hasselt H. Friesen A. Oh J. Chen Y. (2021). Introducing symmetries to black box meta reinforcement learning. arXiv preprint: arXiv:2109.10781.

[B100] Köhler J. Klein L. Noé F. (2020). Equivariant flows: exact likelihood generative learning for symmetric densities, in International Conference on Machine Learning (Vienna), 5361–5370.

[B101] Kriegeskorte N. Mur M. Bandettini P. (2008). Representational similarity analysis - connecting the branches of systems neuroscience. Front. Syst. Neurosci. 2, 1662–5137. 10.3389/neuro.06.004.2008 19104670 PMC2605405

[B102] Krizhevsky A. Sutskever I. Hinton G. E. (2012). Imagenet classification with deep convolutional neural networks, in NIPS (Lake Tahoe).

[B103] Kulkarni T. Whitney W. Kohli P. Tenenbaum J. (2015). Deep convolutional inverse graphics network, in NIPS (Montreal, QC).

[B104] Kumar A. Sattigeri P. Balakrishnan A. (2018). Variational inference of disentangled latent concepts from unlabeled observations, in Proceedings of the Sixth Annual International Conference on Learning Representations (ICLR) (Vancouver, BC).

[B105] Lake B. M. Ullman T. D. Tenenbaum J. B. Gershman S. J. (2016). Building machines that learn and think like people. Behav. Brain Sci. 1–101. 10.1017/S0140525X16001837 27881212

[B106] Laversanne-Finot A. Pere A. Oudeyer P. -Y. (2018). Curiosity driven exploration of learned disentangled goal spaces, in Conference on Robot Learning (PMLR), 487–504.

[B107] LeCun Y. Bengio Y. (1995). Convolutional networks for images, speech, and time series, in The handbook of Brain Theory and Neural Networks (Cambridge, MA), 3361.

[B108] LeCun Y. Boser B. Denker J. S. Henderson D. Howard R. E. Hubbard W.. (1989). Backpropagation applied to handwritten zip code recognition. Neural Comput. 1, 541–551. 10.1162/neco.1989.1.4.541

[B109] Lee W. Kim D. Hong S. Lee H. (2020). High-fidelity synthesis with disentangled representation. arxiv. 10.1007/978-3-030-58574-7_10

[B110] Leibo J. Z. Liao Q. Anselmi F. Freiwald W. A. Poggio T. (2017). View-tolerant face recognition and hebbian learning imply mirror-symmetric neural tuning to head orientation. Curr. Biol. 27, 62–67. 10.1016/j.cub.2016.10.015 27916522 PMC5319833

[B111] Leong Y. C. Radulescu A. Daniel R. DeWoskin V. Niv Y. (2017). Dynamic interaction between reinforcement learning and attention in multidimensional environments. Neuron 93, 451–463. 10.1016/j.neuron.2016.12.040 28103483 PMC5287409

[B112] Liao Q. Leibo J. Z. Poggio T. (2013). Learning invariant representations and applications to face verification, in Advances in Neural Information Processing Systems, eds Burges C. J. C. Bottou L. Welling M. Ghahramani Z. Weinberger K. Q. (Curran Associates). Available online at: https://proceedings.neurips.cc/paper/2013/file/ad3019b856147c17e82a5bead782d2a8-Paper.pdf

[B113] Livio M. (2012). Why symmetry matters. Nature 490, 472–473. 10.1038/490472a 23099384

[B114] Locatello F. Bauer S. Lucic M. Gelly S. Schölkopf B. Bachem O. (2019). Challenging common assumptions in the unsupervised learning of disentangled representations. ICML 97, 4114–4124.

[B115] Locatello F. Poole B. Rätsch G. Schölkopf B. Bachem O. Tschannen M. (2020). Weakly-supervised disentanglement without compromises, in International Conference on Machine Learning (Vienna), 6348–6359.

[B116] Lorenz D. Bereska L. Milbich T. Ommer B. (2019). Unsupervised part-based disentangling of object shape and appearance, in Proceedings of the IEEE Conference on Computer Vision and Pattern Recognition (CVPR) (Long Beach, CA). 10.1109/CVPR.2019.01121

[B117] Lowe D. G. (1999). Object recognition from local scale-invariant features, in The Proceedings of the Seventh IEEE International Conference on Computer Vision, Vol. 2 (Kerkyra), 1150–1157. 10.1109/ICCV.1999.790410

[B118] MacKay D. J. (1995). Free energy minimisation algorithm for decoding and cryptanalysis. Electron. Lett. 31, 446–447. 10.1049/el:19950331

[B119] MacKay D. J. (2003). Information Theory, Inference and Learning Algorithms. Cambridge, UK: Cambridge University Press.

[B120] Madan G. Anand A. Singla P. (2018). Block-value symmetries in probabilistic graphical models. arXiv preprint arXiv:1807.00643. 10.48550/arXiv.1807.00643

[B121] Marcus G. (2018). Deep learning: a critical appraisal. arXiv:1801.00631. 10.48550/arXiv.1801.00631

[B122] Mathieu E. Rainforth T. Siddharth N. Teh Y. W. (2019). Disentangling disentanglement in variational autoencoders, in Proceedings of the 36th International Conference on Machine Learning (ICML) (Long Beach, CA).

[B123] Mazer J. A. Vinje W. E. McDermott J. Schiller P. H. Gallant J. L. (2002). Spatial frequency and orientation tuning dynamics in area v1. Proc. Natl. Acad. Sci. U.S.A. 99, 1645–1650. 10.1073/pnas.022638499 11818532 PMC122244

[B124] Minxha J. Adolphs R. Fusi S. Mamelak A. N. Rutishauser U. (2020). Flexible recruitment of memory-based choice representations by the human medial frontal cortex. Science. 368, eaba3313. 10.1126/science.aba3313 32586990 PMC7531893

[B125] Mnih V. Kavukcuoglu K. Silver D. S. Rusu A. A. Veness J. Bellemare M. G.. (2015). Human-level control through deep reinforcement learning. Nature 518, 529–533. 10.1038/nature14236 25719670

[B126] Niv Y. (2019). Learning task-state representations. Nat. Neurosci. 22, 1544–1553. 10.1038/s41593-019-0470-8 31551597 PMC7241310

[B127] Niv Y. Daniel R. Geana A. Gershman S. J. Leong Y. C. Radulescu A.. (2015). Reinforcement learning in multidimensional environments relies on attention mechanisms. J. Neurosci. 35, 8145–8157. 10.1523/JNEUROSCI.2978-14.2015 26019331 PMC4444538

[B128] Noether E. (1915). The finiteness theorem for invariants of finite groups. Math. Ann. 77, 89–92. 10.1007/BF01456821

[B129] Panichello M. F. Buschman T. J. (2021). Shared mechanisms underlie the control of working memory and attention. Nature 592, 601–605. 10.1038/s41586-021-03390-w 33790467 PMC8223505

[B130] Papamakrios G. Nalisnick E. Rezende D. J. Mohamed S. Lakshminarayanan B. (2021). Normalizing flows for probabilistic modeling and inference, in Journal of Machine Learning Research, 22, 1–64. 32200210

[B131] Pfau D. Higgins I. Botev A. Racaniére S. (2020a). Disentangling by subspace diffusion, in Advances in Neural Information Processing Systems (NeurIPS).

[B132] Pfau D. Spencer J. S. Matthews A. G. Foulkes W. M. C. (2020b). *Ab initio* solution of the many-electron Schrödinger equation with deep neural networks. Phys. Rev. Res. 2:033429. 10.1103/PhysRevResearch.2.033429

[B133] Poggio T. Bizzi E. (2004). Generalization in vision and motor control. Nature 431, 768–774. 10.1038/nature03014 15483597

[B134] Qi C. R. Su H. Mo K. Guibas L. J. (2017). Pointnet: deep learning on point sets for 3d classification and segmentation, in Proceedings of the IEEE Conference on Computer Vision and Pattern Recognition (Honolulu), 652–660.

[B135] Quessard R. Barrett T. D. Clements W. R. (2020). Learning group structure and disentangled representations of dynamical environments. arXiv preprint arXiv:2002.06991. 10.48550/arXiv.2002.06991

[B136] Ramesh A. Choi Y. LeCun Y. (2019). A spectral regularizer for unsupervised disentanglement, in Proceedings of the 36th International Conference on Machine Learning (ICML) (Long Beach, CA).

[B137] Rao R. P. Ballard D. H. (1999). Predictive coding in the visual cortex: a functional interpretation of some extra-classical receptive-field effects. Nat. Neurosci. 2, 79–87. 10.1038/4580 10195184

[B138] Reed S. Sohn K. Zhang Y. Lee H. (2014). Learning to disentangle factors of variation with manifold interaction, in ICML (Beijing).

[B139] Rezende D. J. Mohamed S. Wierstra D. (2014). Stochastic backpropagation and approximate inference in deep generative models. ICML (Beijing), 32, 1278–1286.

[B140] Rezende D. J. Papamakarios G. Racaniére S. Albergo M. Kanwar G. Shanahan P.. (2020). Normalizing flows on tori and spheres, in International Conference on Machine Learning, 8083–8092.

[B141] Rezende D. J. Racaniére S. (2021). Implicit riemannian concave potential maps. arXiv preprint arXiv:2110.01288. 10.48550/arXiv.2110.01288

[B142] Rezende D. J. Racaniére S. Higgins I. Toth P. (2019). Equivariant hamiltonian flows. arXiv preprint arXiv:1909.13739. 10.48550/arXiv.1909.13739

[B143] Ridgeway K. Mozer M. C. (2018). Learning deep disentangled embeddings with the F-statistic loss, in Advances in Neural Information Processing Systems (NeurIPS) (Montreal, QC).

[B144] Rodgers C. C. Nogueira R. Pil B. C. Greeman E. A. Park J. M. Hong Y. K.. (2021). Sensorimotor strategies and neuronal representations for shape discrimination. Neuron 109, 2308–2325. 10.1016/j.neuron.2021.05.019 34133944 PMC8298290

[B145] Rolinek M. Zietlow D. Martius G. (2019). Variational autoencoders pursue PCA directions (by accident), in Proceedings of the IEEE/CVF Conference on Computer Vision and Pattern Recognition (Long Beach, CA), 12406–12415. 10.1109/CVPR.2019.01269

[B146] Rosenblatt F. (1958). The perceptron: a probabilistic model for information storage and organization in the brain. Psychol. Rev. 65:386. 10.1037/h0042519 13602029

[B147] Satorras V. C. Hoogeboom E. Welling M. (2021). Equivariant graph neural networks, in International Conference on Machine Learning (PMLR), 9323–9332.

[B148] Saxena S. Cunningham J. (2019). Towards the neural population doctrine. Curr. Opin. Neurobiol. 55, 103–111. 10.1016/j.conb.2019.02.002 30877963

[B149] Schmidhuber J. (1992). Learning factorial codes by predictability minimization. Neural Comput. 4, 863–869. 10.1162/neco.1992.4.6.863

[B150] Schmidhuber J. (2010). Formal theory of creativity, fun, and intrinsic motivation (1990-2010). IEEE Trans. Auton. Mental Dev. 2, 230–247. 10.1109/TAMD.2010.2056368

[B151] She L. Benna M. K. Shi Y. Fusi S. Tsao D. Y. (2021). The neural code for face memory. bioRxiv [Preprint]. 10.1101/2021.03.12.435023

[B152] Silver D. Huang A. Maddison C. J. Guez A. Sifre L. van den Driessche G.. (2016). Mastering the game of Go with deep neural networks and tree search. Nature 529, 484–489. 10.1038/nature16961 26819042

[B153] Slone L. K. Smith L. B. Yu C. (2019). Self-generated variability in object images predicts vocabulary growth. Dev. Sci. 22:e12816. 10.1111/desc.12816 30770597 PMC6697249

[B154] Smith L. B. Jayaraman S. Clerkin E. Yu C. (2018). The developing infant creates a curriculum for statistical learning. Trends Cogn. Sci. 22, 325–336. 10.1016/j.tics.2018.02.004 29519675 PMC5866780

[B155] Soatto S. (2010). Steps Toward a Theory of Visual Information. Technical Report UCLA-CSD100028 (UCLA).

[B156] Solomonoff R. J. (1964). A formal theory of inductive inference. Part I. Inform. Control 7, 1–22. 10.1016/S0019-9958(64)90223-2

[B157] Soulos P. Isik L. (2020). Disentangled face representations in deep generative models and the human brain, in NeurIPS 2020 Workshop SVRHM.

[B158] Srinivasan M. V. Laughlin S. B. Dubs A. (1982). Predictive coding: a fresh view of inhibition in the retina. Proc. R. Soc. Lond. Ser. B Biol. Sci. 216, 427–459. 10.1098/rspb.1982.0085 6129637

[B159] Stankiewicz B. J. (2002). Empirical evidence for independent dimensions in the visual representation of three-dimensional shape. J. Exp. Psychol. 28:913. 10.1037/0096-1523.28.4.913 12190258

[B160] Steenbrugge X. Leroux S. Verbelen T. Dhoedt B. (2018). Improving generalization for abstract reasoning tasks using disentangled feature representations. arXiv:1811.04784. 10.48550/arXiv.1811.04784

[B161] Sundaramoorthi G. Petersen P. Varadarajan V. S. Soatto S. (2009). On the set of images modulo viewpoint and contrast changes, in 2009 IEEE Conference on Computer Vision and Pattern Recognition (Miami), 832–839. 10.1109/CVPR.2009.5206704

[B162] Tanaka K. (1996). Inferotemporal cortex and object vision. Annu. Rev. Neurosci. 19, 109–139. 10.1146/annurev.ne.19.030196.000545 8833438

[B163] Tang Y. Salakhutdinov R. Hinton G. (2013). Tensor analyzers, in Proceedings of the 30th International Conference on Machine Learning, 2013 (Atlanta, GA).

[B164] Tegmark M. (2008). The mathematical universe. Found. Phys. 38, 101–150. 10.1007/s10701-007-9186-9

[B165] Tenenbaum J. B. De Silva V. Langford J. C. (2000). A global geometric framework for nonlinear dimensionality reduction. Science 290, 2319–2323. 10.1126/science.290.5500.2319 11125149

[B166] Thompson J. A. F. Bengio Y. Formisano E. Schönwiesner M. (2016). How can deep learning advance computational modeling of sensory information processing?, in NeurIPS Workshop on Representation Learning in Artificial and Biological Neural Networks (Barcelona).

[B167] Tishby N. Pereira F. C. Bialek W. (1999). The information bottleneck method, in Proceedings of the 37th Annual Allerton Conference on Communication, Control and Computing (Monticello, IL), 368–377.

[B168] Tishby N. Zaslavsky N. (2015). Deep learning and the information bottleneck principle, in 2015 IEEE Information Theory Workshop (ITW) (Jeju island), 1–5. 10.1109/ITW.2015.7133169

[B169] van der Pol E. Worrall D. van Hoof H. Oliehoek F. Welling M. (2020). MDP homomorphic networks: Group symmetries in reinforcement learning, in Advances in Neural Information Processing Systems, 33.

[B170] van Steenkiste S. Locatello F. Schmidhuber J. Bachem O. (2019). Are disentangled representations helpful for abstract visual reasoning?, in Advances in Neural Information Processing Systems, 32.

[B171] Vaswani A. Shazeer N. Parmar N. Uszkoreit J. Jones L. Gomez A. N.. (2017). Attention is all you need, in Advances in Neural Information Processing Systems (Long Beach, CA), 5998–6008.

[B172] Veeling B. S. Linmans J. Winkens J. Cohen T. Welling M. (2018). Rotation equivariant CNNs for digital pathology, in International Conference on Medical Image Computing and Computer-Assisted Intervention (Granada: Springer), 210–218. 10.1007/978-3-030-00934-2_24

[B173] Wallace C. S. Dowe D. L. (1999). Minimum message length and Kolmogorov complexity. Comput. J. 42, 270–283. 10.1093/comjnl/42.4.270

[B174] Wang J. X. Kurth-Nelson Z. Kumaran D. Tirumala D. Soyer H. Leibo J. Z.. (2018). Prefrontal cortex as a meta-reinforcement learning system. Nat. Neurosci. 21, 860–868. 10.1038/s41593-018-0147-8 29760527

[B175] Wang T. Yue Z. Huang J. Sun Q. Zhang H. (2021). Self-supervised learning disentangled group representation as feature, in Thirty-Fifth Conference on Neural Information Processing Systems.

[B176] Whitney W. F. Chang M. Kulkarni T. Tenenbaum J. B. (2016). Understanding visual concepts with continuation learning. arXiv:1602.06822. 10.48550/arXiv.1602.06822

[B177] Wirnsberger P. Ballard A. J. Papamakarios G. Abercrombie S. Racaniére S. Pritzel A.. (2020). Targeted free energy estimation *via* learned mappings. J. Chem. Phys. 153:144112. 10.1063/5.0018903 33086827

[B178] Wood J. N. Wood S. M. W. (2018). The development of invariant object recognition requires visual experience with temporally smooth objects. J. Physiol. 1–16, 1391–1406. 10.1111/cogs.12595 29537108

[B179] Wulfmeier M. Byravan A. Hertweck T. Higgins I. Gupta A. Kulkarni T.. (2021). Representation matters: improving perception and exploration for robotics, in 2021 IEEE International Conference on Robotics and Automation (ICRA) (Xi'an), 6512–6519. 10.1109/ICRA48506.2021.9560733

[B180] Yamins D. L. K. DiCarlo J. J. (2016). Using goal-driven deep learning models to understand sensory cortex. Nat. Neurosci. 19, 356–365. 10.1038/nn.4244 26906502

[B181] Yamins D. L. K. Hong H. Cadieu C. F. Solomon E. A. Seibert D. DiCarlo J. J. (2014). Performance-optimized hierarchical models predict neural responses in higher visual cortex. Proc. Natl. Acad. Sci. U.S.A. 111, 8619–8624. 10.1073/pnas.1403112111 24812127 PMC4060707

[B182] Yang J. Reed S. Yang M.-H. Lee H. (2015). Weakly-supervised disentangling with recurrent transformations for 3d view synthesis, in NIPS (Montreal, QC).

[B183] Yuste R. (2015). From the neuron doctrine to neural networks. Nat. Rev. Neurosci. 16, 487–497. 10.1038/nrn3962 26152865

[B184] Zhu Z. Luo P. Wang X. Tang X. (2014). Multi-view perceptron: a deep model for learning face identity and view representations, in Advances in Neural Information Processing Systems (Montreal, QC), 27.

